# Association between lactate dehydrogenase and the risk of diabetic kidney disease in patients with type 2 diabetes

**DOI:** 10.3389/fendo.2024.1369968

**Published:** 2024-03-19

**Authors:** Linqiao Tang, Qianyu Yang, Rong Ma, Ping Zhou, Cong Peng, Chunpeng Xie, Qiyuan Liang, Tingyu Wu, Wuyu Gao, Haiyan Yu, Guifei Deng, Zhen Dai, Nan Mao, Xiang Xiao

**Affiliations:** ^1^Research Core Facility of West China Hospital, Sichuan University, Chengdu, Sichuan, China; ^2^Department of Nephrology, The First Affiliated Hospital of Chengdu Medical College, Chengdu, China; ^3^Department of Clinical Medicine, Chengdu Medical College, Chengdu, China; ^4^Department of Nephrology, People’s Hospital of Xindu District, Chengdu, China

**Keywords:** type 2 diabetes mellitus, diabetic kidney disease, database research, NHANES, risk factors

## Abstract

**Objective:**

This study aims to investigate the association between lactate dehydrogenase (LDH) and the risk of diabetic kidney disease (DKD) in patients with type 2 diabetes (T2D).

**Methods:**

The study enrolled patients with diagnosis of T2D between 2009 and 2018 from the National Nutrition and Health Examination Survey (NHANES) database. Demographic information, laboratory test, and diagnostic data were collected. Restricted cubic spline (RCS) plots were used to assess the dose-effect relationship between LDH levels and the risk of DKD in patients with T2D. Based on LDH levels, individuals were divided into higher and lower groups using dichotomy, and multivariate logistic regression analysis was conducted to explore the relationship between different LDH levels and the risk of DKD in T2D patients. Stratified analysis was performed to assess the consistency of the result.

**Results:**

A total of 4888 patients were included in the study, with 2976 (60.9%) patients without DKD and 1912 (39.1%) patients with DKD. RCS plots showed that the risk of DKD increased with increasing LDH levels. Multifactorial logistic regression analysis revealed that T2D patients with higher LDH levels had a 45% increased risk of DKD compared to those with lower LDH levels (OR=1.45; 95% CI: 1.11-1.89). Furthermore, each standard deviation increase in LDH level was associated with a 24% increase in DKD incidence among T2D patients (OR=1.24; 95% CI: 1.07-1.44). Stratified analysis consistently supported these findings.

**Conclusions:**

LDH can serve as a valuable biomarker for screening DKD in patients with T2D.

## 1 Introduction

Diabetes mellitus (DM) is a chronic metabolic disease characterized by impaired insulin secretion and function. It was considered irreversible and has become one of the leading causes of death worldwide (1, 2). The International Diabetes Federation (IDF) predicts that the number of diabetic patients will increase from 240 million in 2007 to 380 million in 2025, further rising to 439 million in 2030 (3). Diabetic kidney disease (DKD), found in approximately 40% of diabetic patients, was the main cause of chronic kidney disease (CKD) and end-stage renal disease (ESRD) globally (4, 5). This condition imposes a significant social and economic burden, highlighting the importance of early identification of DKD.

LDH is an enzyme belonging to the family of 2-hydroxy acid oxidoreductases. It plays a crucial role in both anaerobic and aerobic glycolysis, converting pyruvate into lactic acid and nicotinamide adenine dinucleotide (NADH), thereby affecting metabolism (6). LDH was commonly used for diagnosing such as myocardial infarction, vascular injury, tissue injury, advanced sarcoma, and other mesenchymal tumors (7). It has now been found that LDH can be used as a biomarker for the prognosis of diseases such as tumors, metabolism-associated fatty liver disease, and malaria (8–10), and it can also be used as a biomarker for predicting the pathological status of the lungs (11) and for the possibility of DM (12).

Lactate dehydrogenase (LDH) is an enzyme composed of four subtypes, namely LDH-A, LDH-B, LDH-C, and LDH-D. LDH-A is mainly expressed in muscle and heart tissue, and is involved in the production and dehydrogenation of lactate. It promotes glycolysis to produce ATP under aerobic conditions, while converting lactate to pyruvate under hypoxic conditions to maintain intracellular acid-base balance. LDH-B mainly expressed in the liver and kidneys, involved in lactate metabolism and clearance. which participates in the gluconeogenesis pathway by converting lactate into glucose in the liver, and helps clear excess lactate in the kidneys. LDH-C and LDH-D are expressed in embryonic and germ cells, and their functions are not fully understood, but they may be related to cell proliferation and differentiation. Their proportion in the blood is usually 25-35% for LDH-A (LDH-1), 30-40% for LDH-B (LDH-2), 20-30% for LDH-C (LDH-3), and 5-10% for LDH-D. These proportions may have slight variations, and the specific proportions may also be influenced by different laboratories and measurement methods (13). More and more studies found that LDH may be associated with the development of DKD. Al-Rubeaan K et al. (14)found that the progression of DKD was associated with elevated levels of LDH, and elevated uric acid and LDH were associated with microalbuminuria and an increased risk of ESRD. Mohammadi-Karakani et al (15) observed significantly elevated urinary LDH and microalbuminuria levels in diabetic patients compared to healthy individuals. Lee DY et al. (16) found that elevated levels of LDHA were associated with renal insufficiency in DKD patients. LDHA was expressed at high levels in both glomerular and tubular epithelial cells of renal tissues in DKD patients. The decrease in glomerular filtration rate was associated with increased urinary lactate levels and LDHA expression as well as increased fasting blood glucose and glycosylated hemoglobin levels. In addition, LDH has considerable potential value in early warning of rejection in renal transplant recipients, screening for renal disease, and detection of renal injury secondary to hypertension, diabetes mellitus and rheumatoid arthritis (17).

Therefore, LDH activity and expression levels were elevated in DKD and may be involved in the onset and progression of DKD. However, there were no studies that clearly show the relationship between LDH levels and the risk of DKD in Type 2 diabetes (T2D) patients. Therefore, the aim of this study was to explore the relationship between LDH and the risk of DKD in T2D patients.

## 2 Methods

### 2.1 Study design and participants

The data for this study were obtained from the National Nutrition and Health Survey (NHANES) database from 2009 to 2018, which includes information on patients with T2D. NHANES aims to explore the individual-level demographic, health, and nutritional information through personal interviews and standardized physical exams at specialized Mobile Examination Centers (MECs), as well as evaluate the health and nutritional status of non-hospitalized civilians in the United States (18).

In this study, a total of 4,888 patients were included, consisting of 2976 (60.9%) T2D patients without DKD and 1912 (39.1%) T2D patients with DKD. To ensure the accuracy of the study, specific diagnostic criteria were employed to determine the presence of diabetes and DKD. The diagnostic criteria for diabetes included: 1) doctor diagnosis of diabetes, 2) glycated hemoglobin (HbA1c) level > 6.5%, 3) fasting blood glucose level ≥ 7.0 mmol/L, 4) random blood glucose level ≥ 11.1 mmol/L, or 5) random blood glucose level after a two-hour oral glucose tolerance test (OGTT) ≥ 11.1 mmol/L, meet any one of the above (19). Antidiabetic drugs administration was obtained through a questionnaire.

The diagnostic criteria for DKD were based on the diagnosis of diabetes and met the guidelines for CKD established by the working group for kidney disease improving global outcomes (KDIGO). These criteria included a urinary albumin-to-creatinine ratio (ACR) exceeding 30 mg/g or an estimated glomerular filtration rate (eGFR) below 60 ml/min/1.73m^2^ (20). Exclusion criteria for the study were: 1) age under 18 years, 2) pregnancy, 3) type 1 diabetes mellitus, 4) missing lactate dehydrogenase (LDH) data or abnormally high LDH values, and 5) missing diagnoses of T2D or CKD. NHANES is a survey project conducted by the National Center for Health Statistics of the Centers for Disease Control and Prevention (CDC) and approved by the National Center for Health Statistics Institutional Review Board. The study protocol conformed to the ethical standards of the 1964 Declaration of Helsinki and its subsequent amendments and was approved by the National Ethics Review Board for Health Statistics Research. All participants had signed an informed consent form (19).

### 2.2 Statistical analysis

Following the guidelines of the US Centers for Disease Control and Prevention, the study utilized weighted samples and employed stratification and clustering techniques to generate estimates representative of the overall US population. Continuous variables were summarized using means and standard errors, while classified variables were expressed as percentages and standard errors. To assess differences between different groups, weighted t-tests and chi-square tests were used for continuous variables and categorical variables, respectively.

The relationship between LDH levels and the risk of DKD in T2D patients was evaluated using a restricted cubic spline (RCS) plot of complex sampling. Individuals were classified into two groups, higher LDH group and lower LDH group, through dichotomy. Additionally, a complex sampling multivariate linear regression model was established to evaluate the correlation between LDH and clinical variables after adjusting for multiple factors. Furthermore, a weighted multivariate Cox regression model analysis was performed to identify risk factors for DKD in T2D patients, and the consistency of the results was evaluated through subgroup analysis. Weights were calculated by determining the smallest subset of variables based on their inclusion in the study and selecting the corresponding weights. Finally, the weights were combined over the years. All statistical analyses were performed with R version 4.3.1 software. A significance level of P<0.05 was used for all statistical analyses.

## 3 Results

### 3.1 Baseline characteristics

**Figure 1:**
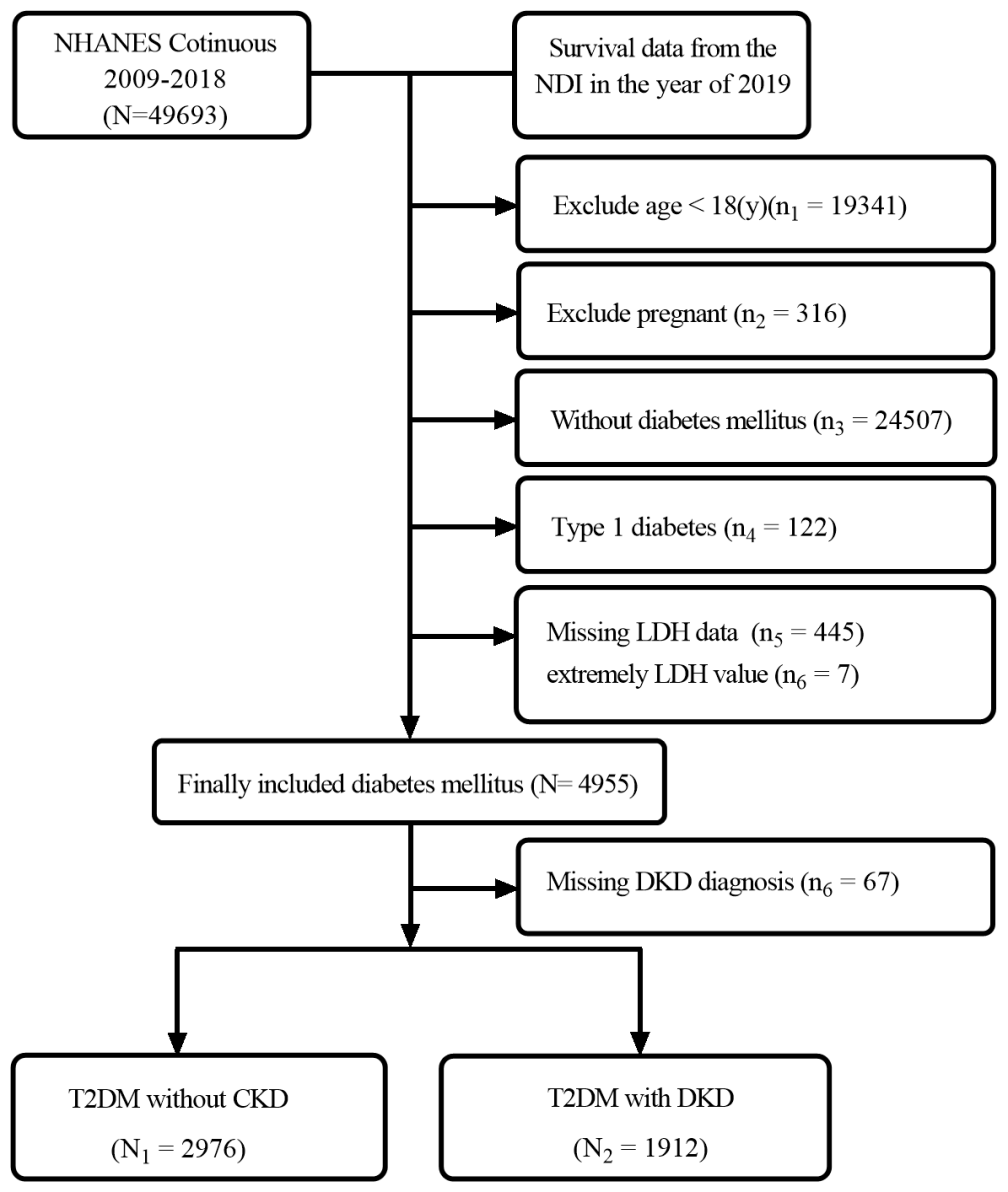
Flowchart of included individuals in this study.

In our study, we utilized the data of 49,693 individuals registered in the NHANES database from 2009 to 2018. In this dataset, we included 4,888 patients diagnosed with T2D (**Figure 1**). The patients enrolled were both male and female, the percentage of males was 51.58. And the average age of T2D patients was 59.59 years. The mean level of LDH was 138.85 U/L. When comparing T2D patients without DKD, we observed that those with DKD were older and had a higher proportion of hypertension and smoking, as well as a lower proportion of drinkers (All P<0.05). Additionally, T2D patients with DKD showed lower eGFR, higher levels of LDH, uric acid, triglyceride, glycosylated hemoglobin, and ACR, and lower levels of hemoglobin and serum albumin (All P<0.05) (**Table 1**). Among the use of antidiabetic drugs, 43.73% of the patients used the oral hypoglycemic agents, 10.87% of the patients used insulin while 6.04% of the patients used each of them. Furthermore, compared with patients with lower LDH, patients with higher LDH were older, had a higher prevalence of women and hypertension, had lower rates of alcohol use and smoking, higher levels of total cholesterol and high-density lipoprotein (HDL), and lower levels of hemoglobin, eGFR, and triglycerides. (all P<0.05) (**Table 2**).

**Table 1:** Baseline clinical features of enrolled T2D patients without and with CKD.

Variable	Total	T2DM without CKD (N1 = 2976)	T2DM with CKD (N2 = 1909)	P-value
LDH (U/L)	138.85 (0.92)	135.01 (0.92)	145.74 (1.54)	< 0.001
Age (years)	59.59 (0.32)	56.52 (0.37)	65.10 (0.45)	< 0.001
BMI (kg/m^2^)	33.16 (0.18)	33.21 (0.21)	33.05 (0.29)	0.62
Hemoglobin (g/dL)	14.00 (0.03)	14.17 (0.04)	13.69 (0.06)	< 0.001
Serum albumin (g/L)	41.38 (0.08)	41.77 (0.10)	40.67 (0.13)	< 0.001
Uric_acid (μmol/L)	341.41 (2.06)	327.10 (2.21)	367.06 (2.98)	< 0.001
Total cholesterol (mmol/L)	4.76 (0.02)	4.78 (0.03)	4.71 (0.03)	0.08
Triglyceride (mmol/L)	2.22 (0.04)	2.15 (0.06)	2.34 (0.05)	0.01
HDL (mmol/L)	1.23 (0.01)	1.22 (0.01)	1.24 (0.01)	0.48
LDL (mmol/L)	2.72 (0.03)	2.76 (0.04)	2.65 (0.04)	0.06
CRP	0.57 (0.04)	0.55 (0.05)	0.61 (0.05)	0.39
eGFR (ml/min/1.73m^2^)	83.60 (0.44)	92.15 (0.45)	68.26 (0.96)	< 0.001
ACR (mg/g)	110.28 (8.45)	10.05 (0.14)	295.64 (24.44)	< 0.001
HbA1c (%)	7.08 (0.03)	6.91 (0.04)	7.38 (0.05)	< 0.001
Sex (%)				0.62
Female	48.42 (0.02)	48.02 (1.55)	49.13 (1.58)	
Male	51.58 (0.02)	51.98 (1.55)	50.87 (1.58)	
Race (%)				0.11
Mexican American	10.30 (0.01)	10.65 (1.28)	9.68 (1.29)	
Non-Hispanic Black	13.62 (0.01)	13.36 (1.19)	14.08 (1.25)	
Non-Hispanic White	60.15 (0.03)	59.34 (2.01)	61.60 (2.00)	
Other Hispanic	6.23 (0.01)	6.82 (0.70)	5.18 (0.70)	
Other Race - Including Multi-Racial	9.70 (0.01)	9.83 (0.81)	9.46 (0.91)	
Hypertension (%)				< 0.001
No	29.73 (0.01)	35.92 (1.36)	18.64 (1.30)	
Yes	70.27 (0.02)	64.08 (1.36)	81.36 (1.30)	
Alcohol user (%)				< 0.001
No	13.18 (0.01)	13.21 (0.78)	17.76 (1.27)	
Yes	75.82 (0.03)	86.79 (0.78)	82.24 (1.27)	
Smoke (%)				0.04
No	50.00 (0.02)	51.79 (1.36)	47.07 (1.64)	
Yes	49.81 (0.02)	48.21 (1.36)	52.93 (1.64)	
Antidiabetic drugs (%)				< 0.001
OHAS	43.73 (0.02)	76.64 (1.53	67.14 (1.47)	
Insulin	10.87 (0.01)	16.46 (1.35)	19.53 (1.39)	
OHAS + Insulin	6.04 (0.00)	6.90 (0.90)	13.32 (1.18)	

BMI, Body Mass Index; ACR, albumin-creatinine ratio; eGFR, estimated glomerular filtration rate; CRP, C-reaction protein; LDH, lactate dehydrogenase; LDL, low density lipoprotein; HDL, high density lipoprotein; OHAS, Oral hypoglycaemic agents; CKD, chronic kidney disease; T2D, type 2 diabetes.

**Table 2:** Baseline clinical features of enrolled T2D patients with various LDH levels.

Variable	Total	Lower LDH (<134U/L) (N1 = 2452)	Higer LDH (≥134U/L) (N2 = 2436)	P-value
LDH (U/L)	138.38 (0.89)	114.74 (0.41)	163.15 (1.09)	< 0.001
Age (years)	59.59 (0.32)	58.32 (0.38)	60.93 (0.46)	< 0.001
BMI (kg/m^2^)	33.16 (0.18)	32.35 (0.20)	34.00 (0.29)	< 0.001
Hemoglobin (g/dL)	14.00 (0.03)	14.07 (0.04)	13.92 (0.05)	0.01
Serum albumin (g/L)	41.38 (0.08)	41.80 (0.09)	40.93 (0.14)	< 0.001
Uric acid (μmol/L)	341.41 (2.06)	336.93 (2.41)	346.10 (3.14)	0.02
Total cholesterol (mmol/L)	4.76 (0.02)	4.72 (0.03)	4.79 (0.04)	0.16
Triglyceride (mmol/L)	2.22 (0.04)	2.26 (0.05)	2.18 (0.07)	0.35
HDL (mmol/L)	1.23 (0.01)	1.19 (0.01)	1.27 (0.01)	< 0.001
LDL (mmol/L)	2.72 (0.03)	2.73 (0.04)	2.71 (0.04)	0.75
CRP	0.57 (0.04)	0.56 (0.04)	0.58 (0.06)	0.58
eGFR (ml/min/1.73m (2))	83.60 (0.44)	86.89 (0.59)	80.15 (0.65)	< 0.001
ACR (mg/g)	110.28 (8.45)	80.19 (10.97)	142.07 (12.68)	< 0.001
HbA1c (%)	7.08 (0.03)	7.08 (0.05)	7.08 (0.04)	0.93
Sex (%)				< 0.001
Female	48.43 (0.02)	45.10 (1.49)	51.92 (1.47)	
Male	51.57 (0.02)	54.90 (1.49)	48.08 (1.47)	
Race (%)				< 0.001
Mexican American	10.30 (0.01)	11.47 (1.55)	9.08 (1.08)	
Non-Hispanic Black	13.61 (0.01)	11.18 (1.01)	16.16 (1.45)	
Non-Hispanic White	60.17 (0.03)	60.97 (2.18)	59.33 (2.01)	
Other Hispanic	6.24 (0.01)	6.86 (0.81)	5.59 (0.64)	
Other Race - Including Multi-Racial	9.67 (0.01)	9.51 (0.91)	9.84 (0.87)	
Hypertension (%)				< 0.001
No	29.70 (0.01)	33.96 (1.51)	25.24 (1.17)	
Yes	70.30 (0.02)	66.04 (1.51)	74.76 (1.17)	
Alcohol user (%)				0.17
No	13.19 (0.01)	13.95 (1.01)	15.79 (0.95)	
Yes	75.81 (0.03)	86.05 (1.01)	84.21 (0.95)	
Smoke (%)				0.22
No	50.04 (0.02)	48.95 (1.25)	51.37 (1.50)	
Yes	49.78 (0.02)	51.05 (1.25)	48.63 (1.50)	
Antidiabetic drugs (%)				< 0.001
OHAS	43.73 (0.02)	76.64 (1.53)	67.14 (1.47)	
Insulin	10.87 (0.01)	16.46 (1.35)	19.53 (1.39)	
OHAS + Insulin	6.04 (0.00)	6.90 (0.90)	13.32 (1.18)	

BMI, Body Mass Index; ACR, albumin-creatinine ratio; eGFR, estimated glomerular filtration rate; CRP, C-reaction protein; LDH, lactate dehydrogenase; LDL, low density lipoprotein; HDL, high density lipoprotein; OHAS, Oral hypoglycaemic agents; CKD, chronic kidney disease; T2D, type 2 diabetes.

### 3.2 Association of LDH with clinical characteristics

After adjusting for age, sex, race, alcohol use (“Yes” or “No”), smoking (“Yes” or “No”), multivariate linear regression model analysis showed that LDH significantly impacted serum albumin, ACR, and eGFR. The level of LDH was positively correlated with urinary ACR (β=1.48, P<0.001) and negatively correlated with serum albumin (β=-0.01, P<0.001) and eGFR (β=-0.06, P<0.001) (**Table 3**).

**Table 3:** Relationship between LDH and clinical indicators in T2D patients.

Variables	Adjusted		
	β	95%CI	p value
Hemoglobin	0.00	0.00 (0.00, 0.00)	0.78
Serum albumin	-0.01	(-0.02, -0.01)	<0.001
Uric acid	0.10	(-0.03, 0.22)	0.14
Total cholesterol	0.00	(0.00, 0.00)	0.01
Triglyceride	0.00	(0.00, 0.01)	0.52
HDL	0.00	(0.00, 0.00)	<0.001
LDL	0.00	(0.00, 0.00)	0.30
CRP	0.00	(0.00, 0.00)	0.13
ACR	1.48	(0.77, 2.18)	<0.001
eGFR	-0.06	(-0.08,-0.03)	<0.001

Adjust for age (<65,≥65), sex (‘Female’, ‘Male’), race, BMI. ACR, albumin-creatinine ratio; eGFR, estimated glomerular filtration rate; BMI, Body Mass Index;LDL, low density lipoprotein; HDL, high density lipoprotein; CRP, C-reaction protein, T2D, type 2 diabetes.

### 3.3 Association between LDH and risk of DKD

**Figure 2:**
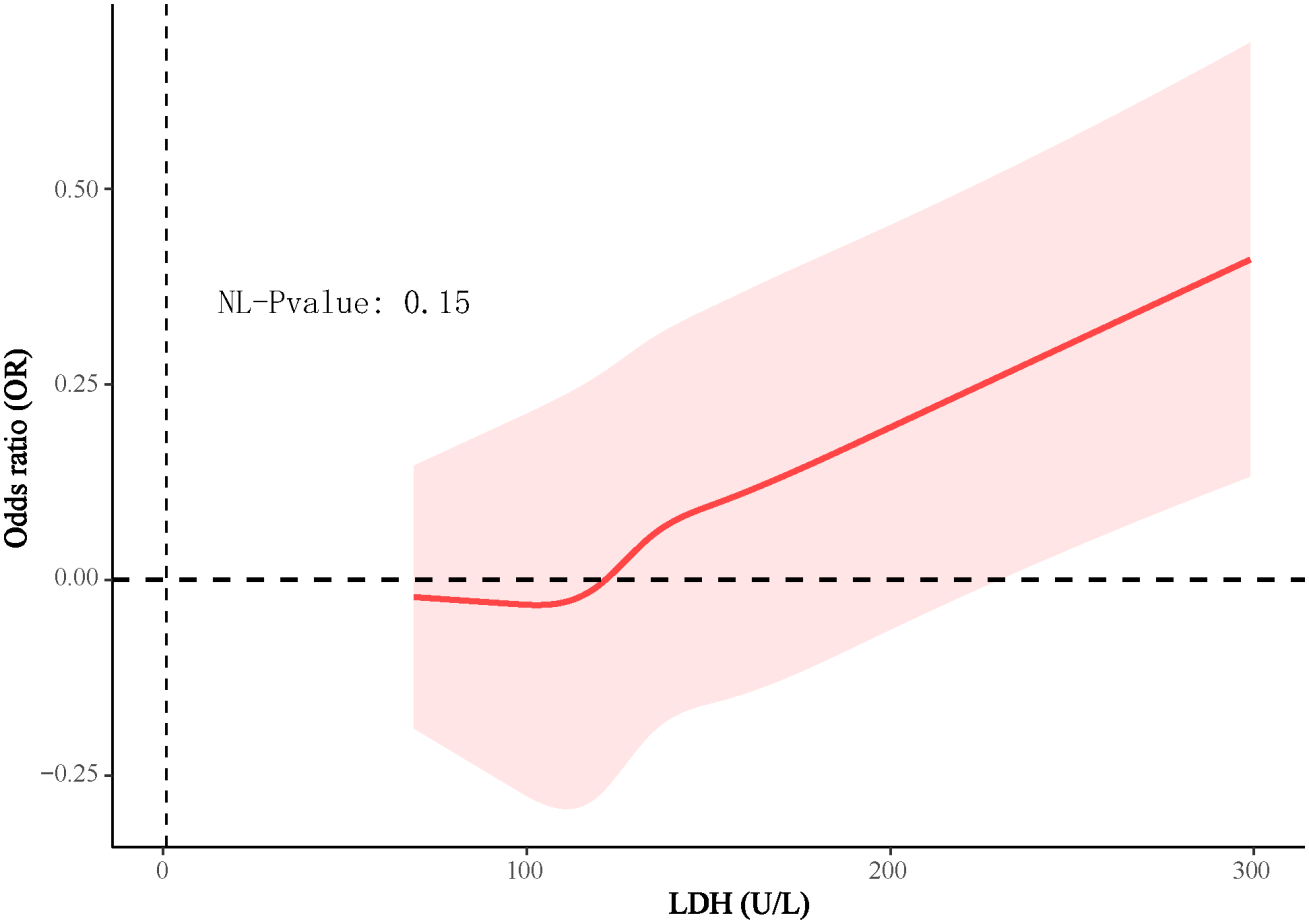
The dose-effect relationship between LDH levels and the risk of DKD in patients with T2D.

**Figure 3:**
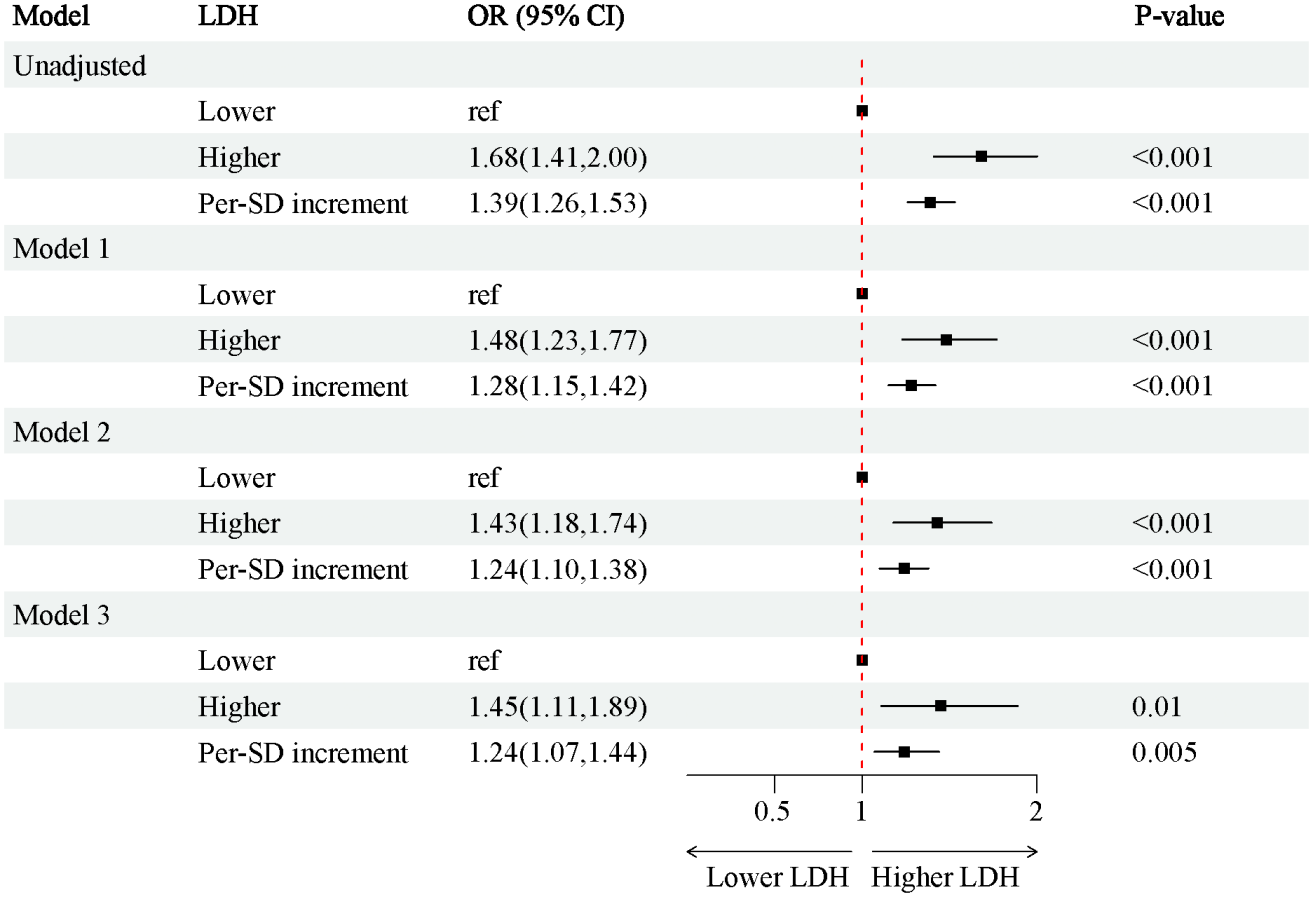
Associations between SII and the risk of CKD in individuals. Model 1^a^ adjusted for baseline age, gender, race; Model 2^b^ adjusted for covariates in model 1 plus alcohol user (‘yes’ or ‘no’), smoke (‘yes’ or ‘no’). Model 3^c^ adjusted for adjusted for covariates in model 2 plus hypertension (‘yes’ or ‘no’), hemoglobin, serum albumin, uric acid. OR, odds ratio; CI, Confidence interval; BMI, Body Mass Index; CRP, C-reaction protein; LDH, lactate dehydrogenase; LDL, low density lipoprotein; HDL, high density lipoprotein; CKD, chronic kidney disease; T2D, type 2 diabetes.

**Figure 4:**
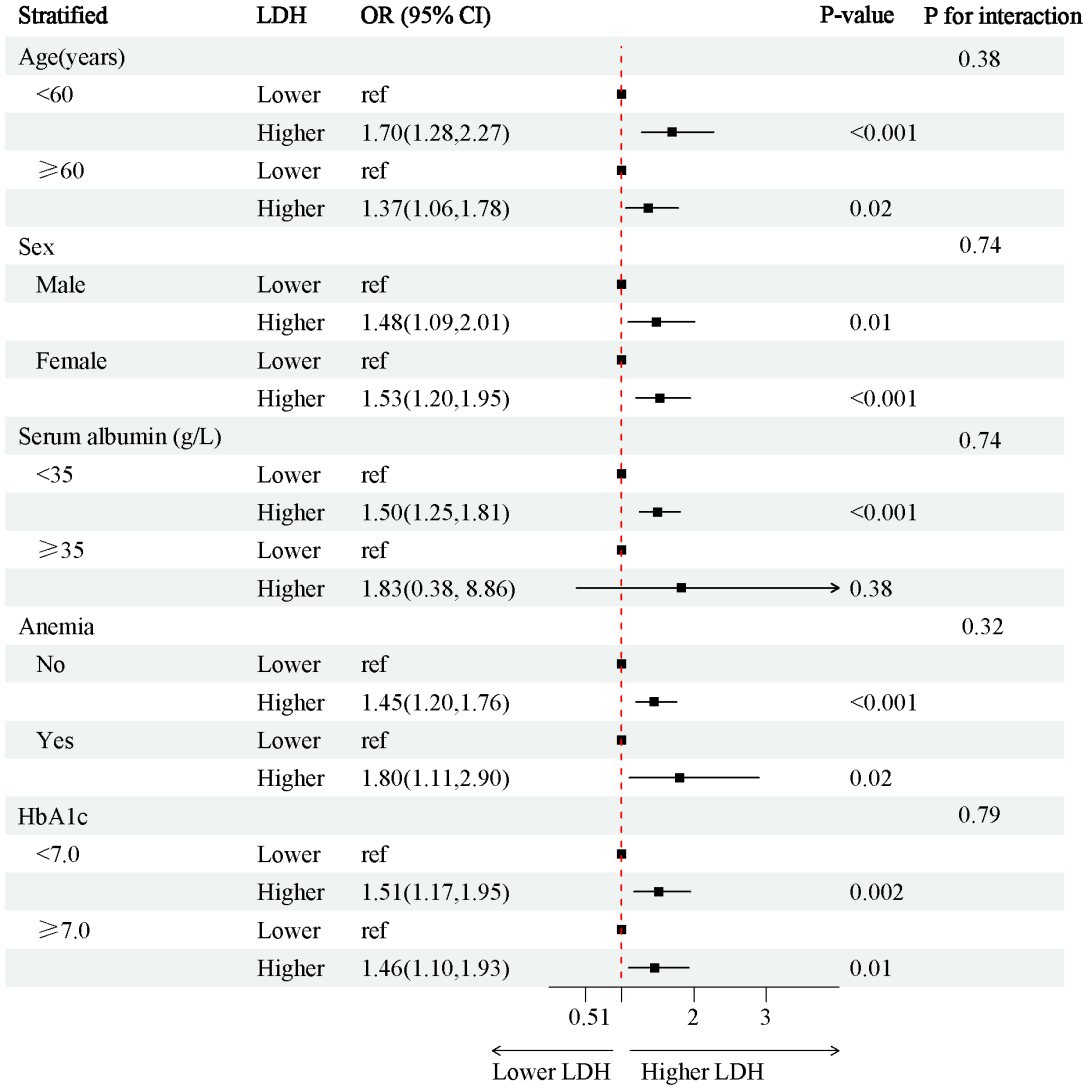
Stratified analysis of the risk of the CKD in individuals with T2D. Adjust for age, sex (‘Female’, ‘Male’), race, BMI. OR, odds ratio; CI, confidence interval; BMI, Body Mass Index; T2D, type 2 diabetes.

In the 4888 T2D patients, 1912 (39.1%) of T2D patients without DKD, whereas 2976 (60.9%) of T2D patients with DKD. The patients were grouped into a lower LDH level group (<134 U/L) (n=2452) and a higher LDH level group (≥134 U/L) (n=2436) based on dichotomization. The results of RCS plots showed a linear relationship between LDH and the risk of DKD in T2D, with the risk of DKD increased in patients with T2D as LDH was elevated (**Figure 2**). Compared with T2D patients with lower LDH, the risk of DKD in T2D with higher LDH (134 < LDH ≤ 367)(U/L) was 39% higher (OR: 1.39; 95% CI, 1.15-1.68, P < 0.001) (**Figure 3**). After adjusting for baseline age, sex, and race, Model 1^a^ revealed that the risk of DKD in T2D patients with higher LDH was 48% higher compared to those with lower LDH (OR = 1.48; 95% CI, 1.23-1.77, P<0.001). Model 2^b^, which included adjustments for covariates from Model 1^a^ as well as alcohol use (“Yes” or “No”) and smoking (“Yes” or “No”), demonstrated that the risk of DKD in T2D patients with higher LDH was 43% higher than those with lower LDH (OR = 1.43; 95% CI, 1.18-1.74, P<0.001). Model 3^c^ was adjusted for covariates from Model 2^b^ as well as hypertension (“Yes” or “No”), hemoglobin, serum albumin, and uric acid. The results indicated that the risk of DKD in T2D patients with higher LDH was 45% higher than those with lower LDH (OR: 1.45; 95% CI, 1.11-1.89, P=0.01) (**Figure 3**; **Supplementary Tables 1**, **2**). Furthermore, each standard deviation (SD) increase in LDH was associated with a 24% increase in the risk of DKD (OR=1.24; 95% CI, 1.07-1.44, P=0.005) (**Figure 3**; **Supplementary Tables 1**, **2**). Stratified analyses showed that the association between LDH and the risk of DKD in patients with T2D varied by age (‘<60’,’≥60’ years), sex (‘male,’ ‘female’), serum albumin (‘<35’,’≥35’ g/L), anemia (‘yes’. ‘No’), and HbA1c (‘<7.0’, ‘≥7.0’) were consistent (all P>0.05) (**Figure 4**).

## 4 Discussion

In this study, we found that LDH was associated with the conventional markers of kidney injury, ACR and eGFR. With increasing levels of LDH, the risk of DKD in T2D patients increased, and higher levels of LDH were an independent risk factor for the risk of DKD in T2D patients.

LDH is an enzyme involved in glycolysis, and there are four isozymes in the human genome: LDHA, LDHB, LDHC, and LDHD. Among these isozymes, LDHA, LDHB and LDHC are L isomers, whereas LDHD is a D isomer (6). The expression of LDH increases with age, and it plays a crucial role in promoting glycolysis by converting pyruvate to lactate and NADH to NAD (21). Numerous studies showed that LDH is closely related to the occurrence, development and prognosis of various tumors. For example, it has been found that elevated expression of LDHA was positively correlated with tumor size, clinical stage, and histological grade, and that its elevated expression was associated with poor prognosis in cancer patients (22, 23). In addition, LDH, as a target of many oncogenes and tumor factors, has shown potential as a therapeutic target for cancer (24).

Moreover, the role of LDH in kidney disease-related mortality has also been reported. Ryu SY et al (25) showed that LDH levels above 280 U/L were positively associated with increased all-cause mortality and cardiovascular mortality in hemodialysis patients, while levels below 240 U/L were associated with improved survival. Shen J et al. (26) found that elevated serum LDH levels were associated to shorter overall survival in renal cell carcinoma patients, suggesting LDH as a valuable biomarker for monitoring prognosis. Zhang D et al (27) found an independent association between LDH levels and in-hospital mortality in patients with acute kidney injury (AKI), where mortality increased with higher LDH levels. Similarly, Xiao X et al. found that high LDH levels were associated with an increased risk of cardiovascular mortality in patients with DKD (28).

Some studies have also found associations between LDH and disease-induced kidney involvement. Cai X et al. (29) demonstrated a positive correlation between LDH and albuminuria severity in hypertensive patients, highlighting high LDH levels as an early marker for increased risk of kidney involvement. Zager RA et al. (30) demonstrated that LDH was released from cells during injury, making it a potential marker for renal injury. In addition, LDH has been considered as a biomarker to predict acute kidney injury in patients with rhabdomyolysis, sickle cell anemia and non-Hodgkin’s lymphoma (31–34). These findings are consistent with that of our study, which showed a significant association between LDH and proteinuria as well as eGFR in patients with T2DM.

In the status of DM, serum LDH levels can be used as a reference marker for short-term glucose monitoring. Changes in serum glycated albumin (GA) and abnormal elevations in LDH levels often occurred simultaneously with the glycemic changes. Therefore, the use of LDH as a biomarker can be more convenient and rapid in assessing glycemic changes in patients with T2D (35). In addition, studies have shown a close relationship between LDH and DKD. Azushima K et al. (36) found that LDH isoenzymes, especially LDHA and LDHB isoenzymes, were increased in patients with DKD. Uslu S et al. (37) found that a significant positive correlation between urinary LDH activity and serum creatinine (Cr) levels, and when creatinine clearance (CCr) decreased, urinary LDH activity began to increase, which suggested that measurement of urinary LDH activity could be used as a screening marker for glomerular and tubular dysfunction in diabetic patients during follow-up. And Jung K et al. (38) found that renal tubular dysfunction can be used as an early indicator of early DKD. These suggested that high LDH levels are associated with early renal function decline in diabetic patients. Xiao X et al (28) found that the risk of progression to ESRD was higher in DKD patients with high levels of LDH. However, few studies have investigated the association between LDH and the risk of DKD in patients with T2D. The association was evidenced in our study, where the risk of DKD in adult T2D patients in the United States increased with elevated LDH, and higher LDH levels were an independent risk factor for the risk of DKD in patients with T2D.

However, why the risk of DKD development is higher in T2D patients with higher LDH needs to be further explored. Firstly, LDH is a catalytic enzyme for lactate formation, and lactate production can contribute to the progression of kidney disease. Lee DY et al. (16) suggested that LDHA-mediated lactic acidosis may be associated with renal failure and fibrosis. Azushima K et al. (36) also conducted targeted metabolomics analysis in a mouse model of DKD and found that LDHA and LDHB isoforms in the DKD mouse was significantly increased in kidneys, and elevated lactate levels and impaired energy metabolism may lead to renal injury in mice. Secondly, Yu SL et al. (39) found that high serum LDH levels were associated with systemic inflammatory responses, and the development of DKD involves inflammatory processes (40). Finally, LDH may influence the lactylation of proteins and consequently the progression of kidney disease (18). Zhang D et al. (41) inhibited lactate production by suppressing LDH activity, and found that intracellular lactate levels and histone lactylation were reduced, and that LDHA-deficient cells had decreased levels of both lactate production and histone lactation levels. Li X et al. (42) found that high lactate concentration and lactylation levels affect energy metabolism, which in turn serves as a predictor of renal injury.

In addition, some of the studies showed that the use of antidiabetic drugs may also affect LDH levels (43). Metformin was shown to decrease LDH activity in cardiomyocytes as well as serum LDH levels (44). In addition, cagliflozin decreased serum LDH levels in diabetic myocardial injury mice. Similar results were observed for other SGLT2i (45, 46). GLP-1/GIP dual agonist has also been shown to decrease serum LDH levels (47). These findings suggest that glucose-lowering drugs may effect LDH levels, but the mechanism is not clear. However, we additionally corrected for the variable of glucose-lowering drug classification in a multivariate logstic regression analysis, which showed that glucose-lowering drug classification did not affect the predictive value of LDH for the risk of CKD in patients with T2DM. Moreover, besides SGLT2i, some studies showed that other renoprotective drugs may also affect LDH (48). Perindopril decreased LDH in serum of mice with diabetic myocardial injury (48). Zahler et al. (49) also found that angiotensin-converting enzyme inhibition (ACEI) attenuated myocardial injury as well as serum levels of LDH. In addition, Ibrahim MA et al. (50) also showed that ACEI and angiotensin AT(1)-receptor antagonism in ameliorating adriamycin-induced cardiotoxicity and nephrotoxicity decreased the level of LDH. However, non-selective saline corticotropin receptor antagonists have not yet been reported to have an effect on LDH. In view of the fact that all these nephroprotective drugs can lower LDH, it is also suggested that LDH plays an important role in kidney injury and hence it can be used to screen patients with DKD in T2DM.

However, we also need to be aware of some limitations of the study. Firstly, due to cross-sectional design, we were unable to establish a cause-effect relationship between LDH levels and the risk of DKD in patients with T2D. We recommend future longer-term follow-up studies to determine the predictive ability of LDH for the occurrence of DKD. Secondly, there was some selection bias due to the retrospective character of the study. Thirdly, although we controlled for some factors that may have influenced the results, there are still some other factors that may have had an impact on the results. Finally, further research is needed on the possible relationship between variations in LDH concentrations and the development of DKD in patients with T2D.

In conclusion, our findings suggest that higher LDH levels were associated with the risk of DKD in T2D patients. It will be necessary for clinicians to monitor LDH levels in patients with T2D, which can assist in screening for DKD in patients with T2D.

## Data availability statement

The datasets presented in this study can be found in online repositories. The names of the repository/repositories and accession number(s) can be found in the article/**Supplementary Material**.

## Ethics statement

The study protocol conformed to the ethical standards of the 1964 Declaration of Helsinki and its subsequent amendments and was approved by the National Ethics Review Board for Health Statistics Research. All participants had signed an informed consent form. The studies were conducted in accordance with the local legislation and institutional requirements. The participants provided their written informed consent to participate in this study. Written informed consent was obtained from the individual(s) for the publication of any potentially identifiable images or data included in this article.

## Author contributions

XX: Writing – review & editing, Conceptualization, Data curation, Formal analysis, Funding acquisition, Investigation, Methodology, Project administration, Resources, Software, Supervision, Validation, Visualization. LT: Writing – original draft, Formal analysis, Resources, Software, Validation. QY: Writing – original draft, Conceptualization, Data curation, Methodology. RM: Writing – original draft. PZ: Writing – original draft. CP: Writing – original draft. CX: Writing – original draft. QL: Writing – original draft. TW: Writing – original draft. WG: Writing – original draft. HY: Writing – original draft. GD: Writing – original draft. ZD: Writing – original draft. NM: Writing – original draft, Supervision, Validation.

## References

[1] Shakoor H Apostolopoulos V Feehan J Ali HI Ismail LC Al Dhaheri A. Effect of calorie restriction and exercise on type 2 diabetes. Prilozi (Makedonska akademija na naukite i umetnostite Oddelenie za medicinski nauki). (2021) 42:109–26. doi: 10.2478/prilozi-2021-0010 33894117

[2] Reed J Bain S Kanamarlapudi V. A review of current trends with type 2 diabetes epidemiology, aetiology, pathogenesis, treatments and future perspectives. Diabetes Metab syndrome Obes Targets Ther. (2021) 14:3567–602. doi: 10.2147/dmso.S319895 PMC8369920 34413662

[3] Yang L Shao J Bian Y Wu H Shi L Zeng L. Prevalence of type 2 diabetes mellitus among inland residents in China (2000-2014): A meta-analysis. J Diabetes Investig. (2016) 7:845–52. doi: 10.1111/jdi.12514 PMC5089946 27181391

[4] Zhang XX Kong J Yun K. Prevalence of diabetic nephropathy among patients with type 2 diabetes mellitus in China: A meta-analysis of observational studies. J Diabetes Res. (2020) 2020:2315607. doi: 10.1155/2020/2315607 32090116 PMC7023800

[5] Alicic RZ Rooney MT Tuttle KR. Diabetic kidney disease: challenges, progress, and possibilities. Clin J Am Soc Nephrol CJASN. (2017) 12:2032–45. doi: 10.2215/cjn.11491116 PMC5718284 28522654

[6] Valvona CJ Fillmore HL Nunn PB Pilkington GJ. The regulation and function of lactate dehydrogenase A: therapeutic potential in brain tumor. Brain Pathol. (2016) 26:3–17. doi: 10.1111/bpa.12299 26269128 PMC8029296

[7] Cassier PA Polivka V Judson I Soria JC Penel N Marsoni S. Outcome of patients with sarcoma and other mesenchymal tumours participating in phase I trials: a subset analysis of a European Phase I database. Ann Oncol. (2014) 25:1222–8. doi: 10.1093/annonc/mdu108 24608201

[8] Comandatore A Franczak M Smolenski RT Morelli L Peters GJ Giovannetti E. Lactate Dehydrogenase and its clinical significance in pancreatic and thoracic cancers. Semin Cancer Biol. (2022) 86:93–100. doi: 10.1016/j.semcancer.2022.09.001 36096316

[9] Lv J Zhou Z Wang J Yu H Lu H Yuan B. Prognostic value of lactate dehydrogenase expression in different cancers: A meta-analysis. Am J Med Sci. (2019) 358:412–21. doi: 10.1016/j.amjms.2019.09.012 31813468

[10] Macías-Rodríguez RU Solís-Ortega AA Ornelas-Arroyo VJ Ruiz-Margáin A González-Huezo MS Urdiales-Morán NA. Prognostic performance of an index based on lactic dehydrogenase and transaminases for patients with liver steatosis and COVID-19. World J Gastroenterol. (2022) 28:5444–56. doi: 10.3748/wjg.v28.i37.5444 PMC9611707 36312835

[11] Drent M Cobben NA Henderson RF Wouters EF van Dieijen-Visser M. Usefulness of lactate dehydrogenase and its isoenzymes as indicators of lung damage or inflammation. Eur Respir J. (1996) 9:1736–42. doi: 10.1183/09031936.96.09081736 8866602

[12] Wang Z Nielsen PM Laustsen C Bertelsen LB. Metabolic consequences of lactate dehydrogenase inhibition by oxamate in hyperglycemic proximal tubular cells. Exp Cell Res. (2019) 378:51–6. doi: 10.1016/j.yexcr.2019.03.001 30836064

[13] Osis G Traylor AM Black LM Spangler D George JF Zarjou A. Expression of lactate dehydrogenase A and B isoforms in the mouse kidney. Am J Physiol Renal Physiol. (2021) 320:F706–f18. doi: 10.1152/ajprenal.00628.2020 PMC8424554 33719570

[14] Al-Rubeaan K Siddiqui K Alghonaim M Youssef AM AlNaqeb D. The Saudi Diabetic Kidney Disease study (Saudi-DKD): clinical characteristics and biochemical parameters. Ann Saudi Med. (2018) 38:46–56. doi: 10.5144/0256-4947.2018.03.01.1010 29295969 PMC6074186

[15] Mohammadi-Karakani A Asgharzadeh-Haghighi S Ghazi-Khansari M Hosseini R. Determination of urinary enzymes as a marker of early renal damage in diabetic patients. J Clin Lab Anal. (2007) 21:413–7. doi: 10.1002/jcla.20212 PMC6649252 18022929

[16] Lee DY Kim JY Ahn E Hyeon JS Kim GH Park KJ. Associations between local acidosis induced by renal LDHA and renal fibrosis and mitochondrial abnormalities in patients with diabetic kidney disease. Trans Res J Lab Clin Med. (2022) 249:88–109. doi: 10.1016/j.trsl.2022.06.015 35788054

[17] Price RG. Urinary enzymes, nephrotoxicity and renal disease. Toxicology. (1982) 23:99–134. doi: 10.1016/0300-483x(82)90092-0 6126019

[18] Archer E Pavela G Lavie CJ. The inadmissibility of what we eat in america and NHANES dietary data in nutrition and obesity research and the scientific formulation of national dietary guidelines. Mayo Clinic Proc. (2015) 90:911–26. doi: 10.1016/j.mayocp.2015.04.009 PMC4527547 26071068

[19] Ma R Xie C Wang S Xiao X. Retinol intake is associated with the risk of chronic kidney disease in individuals with type 2 diabetes mellitus: results from NHANES. Sci Rep. (2023) 13:11567. doi: 10.1038/s41598-023-38582-z 37463986 PMC10354112

[20] Kang H Lee JP Choi K. Exposure to phthalates and environmental phenols in association with chronic kidney disease (CKD) among the general US population participating in multi-cycle NHANES (2005-2016). Sci total Environ. (2021) 791:148343. doi: 10.1016/j.scitotenv.2021.148343 34126474

[21] Hunt LC Demontis F. Age-related increase in lactate dehydrogenase activity in skeletal muscle reduces life span in drosophila. journals gerontology Ser A Biol Sci Med Sci. (2022) 77:259–67. doi: 10.1093/gerona/glab260 PMC8824701 34477202

[22] Girgis H Masui O White NM Scorilas A Rotondo F Seivwright A. Lactate dehydrogenase A is a potential prognostic marker in clear cell renal cell carcinoma. Mol Cancer. (2014) 13:101. doi: 10.1186/1476-4598-13-101 24885701 PMC4022787

[23] Brand A Singer K Koehl GE Kolitzus M Schoenhammer G Thiel A. LDHA-associated lactic acid production blunts tumor immunosurveillance by T and NK cells. Cell Metab. (2016) 24:657–71. doi: 10.1016/j.cmet.2016.08.011 27641098

[24] Ye Y Chen M Chen X Xiao J Liao L Lin F. Clinical significance and prognostic value of lactate dehydrogenase expression in cervical cancer. Genet testing Mol Biomarkers. (2022) 26:107–17. doi: 10.1089/gtmb.2021.0006 PMC8982136 35349377

[25] Ryu SY Kleine CE Hsiung JT Park C Rhee CM Moradi H. Association of lactate dehydrogenase with mortality in incident hemodialysis patients. Nephrology dialysis Transplant. (2021) 36:704–12. doi: 10.1093/ndt/gfaa277 PMC8599773 33367881

[26] Shen J Chen Z Zhuang Q Fan M Ding T Lu H. Prognostic value of serum lactate dehydrogenase in renal cell carcinoma: A systematic review and meta-analysis. PloS One. (2016) 11:e0166482. doi: 10.1371/journal.pone.0166482 27861542 PMC5115746

[27] Zhang D Shi L. Serum lactate dehydrogenase level is associated with in-hospital mortality in critically Ill patients with acute kidney injury. Int Urol Nephrol. (2021) 53:2341–48. doi: 10.1007/s11255-021-02792-z PMC7883888 33590453

[28] Xiao X Zhang J Lang Y Cai L Yang Q Liu K. Associations of lactate dehydrogenase with risk of renal outcomes and cardiovascular mortality in individuals with diabetic kidney disease. Diabetes Res Clin Pract. (2023) 203:110838. doi: 10.1016/j.diabres.2023.110838 37478980

[29] Cai X Wang T Ye C Xu G Xie L. Relationship between lactate dehydrogenase and albuminuria in Chinese hypertensive patients. J Clin hypertension (Greenwich Conn). (2021) 23:128–36. doi: 10.1111/jch.14118 PMC8030071 33283950

[30] Zager RA Johnson AC Becker K. Renal cortical lactate dehydrogenase: A useful, accurate, quantitative marker of *in vivo* tubular injury and acute renal failure. PloS One. (2013) 8:e66776. doi: 10.1371/journal.pone.0066776 23825563 PMC3689004

[31] Heidari Beigvand H Heidari K Hashemi B Saberinia A. The value of lactate dehydrogenase in predicting rhabdomyolysis-induced acute renal failure; a narrative review. Arch Acad Emergency Med. (2021) 9:e24. doi: 10.22037/aaem.v9i1.1096 PMC8126348 34027419

[32] Alzahri MS Mousa SA Almomen AM Hasanato RM Polimeni JM Racz MJ. Lactate dehydrogenase as a biomarker for early renal damage in patients with sickle cell disease. Saudi J Kidney Dis Transplant. (2015) 26:1161–8. doi: 10.4103/1319-2442.168596 26586054

[33] Saraf SL Zhang X Kanias T Lash JP Molokie RE Oza B. Haemoglobinuria is associated with chronic kidney disease and its progression in patients with sickle cell anaemia. Br J haematology. (2014) 164:729–39. doi: 10.1111/bjh.12690 PMC3945021 24329963

[34] Pedersen LM Sørensen PG. Clinical significance of urinary albumin excretion in patients with non-Hodgkin's lymphoma. Br J haematology. (1999) 107:889–91. doi: 10.1046/j.1365-2141.1999.01772.x 10606899

[35] Hsieh YS Yeh MC Lin YY Weng SF Hsu CH Huang CL. Is the level of serum lactate dehydrogenase a potential biomarker for glucose monitoring with type 2 diabetes mellitus? Front Endocrinol. (2022) 13:1099805. doi: 10.3389/fendo.2022.1099805 PMC9801409 36589820

[36] Azushima K Kovalik JP Yamaji T Ching J Chng TW Guo J. Abnormal lactate metabolism is linked to albuminuria and kidney injury in diabetic nephropathy. Kidney Int. (2023) 104:1135–49. doi: 10.1016/j.kint.2023.08.006 37843477

[37] Uslu S Efe B Alataş O Kebapçi N Colak O Demirüstü C. Serum cystatin C and urinary enzymes as screening markers of renal dysfunction in diabetic patients. J Nephrol. (2005) 18:559–67. 16299682

[38] Jung K Pergande M Schimke E Ratzmann KP. [Urine enzymes and low molecular weight proteins as indicators of diabetic nephropathy]. Klinische Wochenschrift. (1989) 67 Suppl 17:27–30. 2739355

[39] Yu SL Xu LT Qi Q Geng YW Chen H Meng ZQ. Serum lactate dehydrogenase predicts prognosis and correlates with systemic inflammatory response in patients with advanced pancreatic cancer after gemcitabine-based chemotherapy. Sci Rep. (2017) 7:45194. doi: 10.1038/srep45194 28345594 PMC5366928

[40] Zheng W Guo J Liu ZS. Effects of metabolic memory on inflammation and fibrosis associated with diabetic kidney disease: an epigenetic perspective. Clin Epigenet. (2021) 13:87. doi: 10.1186/s13148-021-01079-5 PMC8061201 33883002

[41] Zhang D Tang Z Huang H Zhou G Cui C Weng Y. Metabolic regulation of gene expression by histone lactylation. Nature. (2019) 574:575–80. doi: 10.1038/s41586-019-1678-1 PMC6818755 31645732

[42] Li X Yang Y Zhang B Lin X Fu X An Y. Lactate metabolism in human health and disease. Signal transduction targeted Ther. (2022) 7:305. doi: 10.1038/s41392-022-01151-3 PMC9434547 36050306

[43] Zhang J Huang L Shi X Yang L Hua F Ma J. Metformin protects against myocardial ischemia-reperfusion injury and cell pyroptosis via AMPK/NLRP3 inflammasome pathway. Aging. (2020) 12:24270–87. doi: 10.18632/aging.202143 PMC7762510 33232283

[44] Du S Shi H Xiong L Wang P Shi Y. Canagliflozin mitigates ferroptosis and improves myocardial oxidative stress in mice with diabetic cardiomyopathy. Front Endocrinol. (2022) 13:1011669. doi: 10.3389/fendo.2022.1011669 PMC9616119 36313744

[45] Zhang Z Jin X Yang C Li Y. Teneligliptin protects against hypoxia/reoxygenation-induced endothelial cell injury. Biomedicine pharmacotherapy = Biomedecine pharmacotherapie. (2019) 109:468–74. doi: 10.1016/j.biopha.2018.10.016 30399583

[46] Al-Kuraishy HM Al-Gareeb AI Qusty N Alexiou A Batiha GE. Impact of sitagliptin on non-diabetic covid-19 patients. Curr Mol Pharmacol. (2022) 15:683–92. doi: 10.2174/1874467214666210902115650 34477540

[47] Wang Y Cai F Li G Tao Y. Novel dual glucagon-like peptide-1/ glucose-dependent insulinotropic polypeptide receptor agonist attenuates diabetes and myocardial injury through inhibiting hyperglycemia, inflammation and oxidative stress in rodent animals. Bioengineered. (2022) 13:9184–96. doi: 10.1080/21655979.2022.2051859 PMC9161981 35383532

[48] Patel BM Agarwal SS Bhadada SV. Perindopril protects against streptozotocin-induced hyperglycemic myocardial damage/alterations. Hum Exp Toxicol. (2012) 31:1132–43. doi: 10.1177/0960327112446817 22653691

[49] Koszegi S Molnar A Lenart L Hodrea J Balogh DB Lakat T. RAAS inhibitors directly reduce diabetes-induced renal fibrosis via growth factor inhibition. J Physiol. (2019) 597:193–209. doi: 10.1113/jp277002 30324679 PMC6312411

[50] Ibrahim MA Ashour OM Ibrahim YF El-Bitar HI Gomaa W Abdel-Rahim SR. Angiotensin-converting enzyme inhibition and angiotensin AT(1)-receptor antagonism equally improve doxorubicin-induced cardiotoxicity and nephrotoxicity. Pharmacol Res. (2009) 60:373–81. doi: 10.1016/j.phrs.2009.05.007 19467331

