# Supplementary material for: Association between lactate dehydrogenase and the risk of diabetic kidney disease in patients with type 2 diabetes
Source: Front Endocrinol (Lausanne). 2024 Mar 19;15:1369968. doi: 10.3389/fendo.2024.1369968 (PMC10985160; doi:10.3389/fendo.2024.1369968)
Supplement: Supplementary file 1 [file DataSheet_1.docx]

**Supplementary -table 1. Cox-regression analysis of risk factors for development of CKD in T2DM**

| Variables | Unadjusted | | | Model 3 | | |
| --- | --- | --- | --- | --- | --- | --- |
|  | OR | 95%CI | P-value | OR | (95%CI) | P-value |
| LDH |  |  |  |  |  |  |
| Lower (38≤LDH≤134) (U/L) | ref | ref |  | ref | ref |  |
| Higher (134＜LDH≤367)(U/L) | 1.68 | (1.41,2.00) | <0.001 | 1.39 | (1.15,1.68) | 0.001 |
| Age | 1.05 | (1.05,1.06) | <0.001 | 1.05 | (1.04,1.05) | <0.001 |
| Gender |  |  | <0.001 |  |  |  |
| Female | ref | ref |  |  |  |  |
| Male | 0.96 | (0.80,1.14) | 0.62 | 1.08 | (0.86,1.36) | 0.52 |
| BMI | 1.00 | (0.99,1.01) | 0.63 | — | — | — |
| Ethic |  |  |  |  |  |  |
| Mexican American | ref | ref |  | ref | ref |  |
| Non-Hispanic Black | 1.16 | (0.94,1.43) | 0.16 | 0.57 | (0.44,0.75) | <0.001 |
| Non-Hispanic White | 1.14 | (0.94,1.38) | 0.18 | 0.62 | (0.48,0.81) | <0.001 |
| Other Hispanic | 0.83 | (0.65,1.08) | 0.17 | 0.64 | (0.46,0.90) | 0.01 |
| Other Race - Including Multi-Racial | 1.05 | (0.81,1.36) | 0.66 | 0.67 | (0.48,0.93) | 0.02 |
| Hypertension |  |  |  |  |  |  |
| No | ref | ref |  | ref | ref |  |
| Yes | 2.45 | (2.04,2.94) | <0.001 | 1.62 | (1.30,2.01) | <0.001 |
| Alcohol user |  |  |  |  |  |  |
| No | ref | ref |  | ref | ref |  |
| Yes | 0.70 | (0.58,0.86) | <0.001 | 0.76 | (0.61,0.96) | 0.02 |
| Smoking |  |  |  |  |  |  |
| No | ref | ref |  | ref | ref |  |
| Yes | 1.21 | (1.01,1.45) | 0.04 | 1.26 | (1.01,1.58) | 0.05 |
| Hemoglobin | 0.82 | (0.78,0.87) | <0.001 | 0.92 | (0.85,0.99) | 0.03 |
| Serum albumin | 0.91 | (0.89,0.94) | <0.001 | 0.93 | (0.90,0.96) | <0.001 |
| Uric acid | 1.01 | (1.00,1.01) | <0.001 | 1.00 | (1.00,1.01) | <0.001 |
| Triglyceride | 1.05 | (0.99,1.11) | 0.10 | — | — | — |
| Total cholesterol | 0.95 | (0.89,1.01) | 0.09 | — | — | — |
| LDL | 0.89 | (0.80,1.01) | 0.06 | — | — | — |
| HDL | 1.08( | 0.88,1.34) | 0.46 | — | — | — |
| CRP | 1.08 | (0.90,1.30) | 0.39 | — | — | — |

|  |
| --- |

OR, odds ratio; CI, Confidence interval; BMI, Body Mass Index; CRP, C-reaction protein; LDH, lactate dehydrogenase; LDL, low density lipoprotein; HDL, high density lipoprotein

**Supplementary -table 2 . Associations between LDH level and development of CKD in T2D**

| Variables | Unadjusted | | Model 1**^a^** | | Model 2**^b^** | | Model 3**^c^** | |
| --- | --- | --- | --- | --- | --- | --- | --- | --- |
|  | 95%CI | P | 95%CI | P | 95%CI | P | 95%CI | P |
| Lower LDH  (38 ≤ LDH ≤ 134) (U/L) | ref |  | ref |  | ref |  |  |  |
| Higher LDH  (134＜LDH ≤ 367) (U/L) | 1.68(1.41,2.00) | <0.001 | 1.48(1.23,1.77) | <0.001 | 1.43(1.18,1.74) | <0.001 | 1.45(1.11,1.89) | 0.01 |
| Per-SD increment of LDH | 1.39(1.26,1.53) | <0.001 | 1.28(1.15,1.42) | <0.001 | 1.24(1.10,1.38) | <0.001 | 1.24(1.07,1.44) | 0.005 |

**Model 1^a^** adjusted for baseline age, gender, ethic; **Model 2^b^** adjusted for covariates in model 1 plus alcohol user (‘yes’ or ‘no’), smoke (‘yes’ or ‘no’). **Model 3^c^** adjusted for adjusted for covariates in model 2 plus hypertension (‘yes’ or ‘no’), hemoglobin, serum albumin, uric acid. OR, odds ratio; CI, Confidence interval; BMI, Body Mass Index; CRP, C-reaction protein; LDH, lactate dehydrogenase; LDL, low density lipoprotein; HDL, high density lipoprotein; CKD, chronic kidney disease; T2D, type 2 diabetes.
